# Expression of the SARS-CoV-2 receptor-binding domain by live attenuated influenza vaccine virus as a strategy for designing a bivalent vaccine against COVID-19 and influenza

**DOI:** 10.1186/s12985-024-02350-w

**Published:** 2024-04-09

**Authors:** Ekaterina Stepanova, Irina Isakova-Sivak, Daria Mezhenskaya, Sergei Niskanen, Victoria Matyushenko, Ekaterina Bazhenova, Alexandra Rak, Pei Fong Wong, Polina Prokopenko, Tatiana Kotomina, Elena Krutikova, Sergei Legotskiy, Bogdan Neterebskii, Tatiana Ostroukhova, Konstantin Sivak, Yana Orshanskaya, Kirill Yakovlev, Larisa Rudenko

**Affiliations:** 1 Institute of Experimental Medicine, Saint Petersburg, 197022 Russia; 2 Joint-Stock Company «BIOCAD» (JSC «BIOCAD») Saint Petersburg, Intracity Municipality the Settlement of Strelna, the Settlement of Strelna, ul. Svyazi, d. 38, str. 1, pomeshch. 89, Saint Petersburg, 198515 Russia; 3 Smorodintsev Research Institute of Influenza, Saint Petersburg, 197376 Russia

**Keywords:** SARS-CoV-2, COVID-19, Influenza, Bivalent vaccine, Recombinant influenza virus, Virus vectored vaccine, Immunogenicity, Syrian hamsters

## Abstract

Influenza and SARS-CoV-2 are two major respiratory pathogens that cocirculate in humans and cause serious illness with the potential to exacerbate disease in the event of co-infection. To develop a bivalent vaccine, capable of protecting against both infections, we inserted the receptor-binding domain (RBD) of the SARS-CoV-2 spike protein into hemagglutinin (HA) molecule or into the open reading frame of the truncated nonstructural protein 1 (NS1) of live attenuated influenza vaccine (LAIV) virus and assessed phenotypic characteristics of the rescued LAIV-RBD viruses, as well as their immunogenicity in mouse and Syrian hamster animal models. A panel of 9 recombinant LAIV-RBD viruses was rescued using the A/Leningrad/17 backbone. Notably, only two variants with RBD insertions into the HA molecule could express sufficient quantities of RBD protein in infected MDCK cells. Intranasal immunization of mice induced high levels of anti-influenza antibody responses in all chimeric LAIV-RBD viruses, which was comparable to the LAIV virus vector. The RBD-specific antibody responses were most pronounced in the variant expressing RBD194 fragment as a chimeric HA protein. This candidate was further tested in Syrian hamsters and was shown to be immunogenic and capable of protecting animals against both infections.

**Supplementary Information:**

The online version contains supplementary material available at 10.1186/s12985-024-02350-w.

## Introduction

Influenza and SARS-CoV-2 viruses have cocirculated since 2020. Both pathogens have high variability rate, cause millions of cases every year, and can coinfect individuals with increased risk of complications [1, 2]. Annual vaccination is the most effective strategy to control influenza epidemics. The development of a bivalent vaccine against influenza and SARS-CoV-2 is highly relevant since the existing system of annual influenza immunization can be easily adjusted for prophylaxis of both infections.

While there is much interest in this idea, quite a variety of bivalent vaccines are being developed. An adenovirus-based bivalent vaccine encoding SARS-CoV-2 receptor-binding domain (RBD) and H7N9 influenza HA conserved stalk domain protected mice against challenge with influenza and induced neutralizing antibody response to SARS-CoV-2 [3]. Another viral vector-based bivalent vaccine was developed using vesicular stomatitis virus; this vaccine expressed SARS-CoV-2 full-length spike or RBD and influenza M2, and demonstrated promising results in animal studies [4]. Furthermore, influenza VLP-based vaccine with addition of (GPI)-anchored SARS-CoV-2 RBD fused to GM-CSF had protective potential against both viruses [5], and even inactivated influenza virus with the RBD conjugated onto its surface was immunogenic in preclinical studies [6]. Notably, the Moderna’s mRNA-1083 vaccine candidate demonstrated positive results in a phase I/II clinical trial (NCT05827926) [7], shortly after Pfizer and BioNTech mRNA vaccines against influenza and SARS-CoV-2 were reported also to be safe and well-tolerated (NCT05596734) [8].

The design of such bivalent vaccines using influenza virus as a viral vector is an attractive idea, as there is established system with annual influenza vaccinations, and because influenza virus-based vector platforms have been well studied and characterized. The most popular SARS-CoV-2 antigen in influenza-based vaccines is the RBD of the viral spike protein, because of limited capacity of the vector, and proven effectiveness of the RBD as an antigen for COVID-19 vaccines. The bivalent vaccine based on attenuated H1N1pdm virus is being developed by a group of scientists from China [9]. In this development, the RBD is embedded in the NS gene, while the virus lacks the NS1 protein. This vaccine showed promising results in protection studies during preclinical evaluation, even despite the low level of immune response recorded in the neutralization test. Furthermore, this vaccine was proven to be safe, well-tolerated and immunogenic in a phase I clinical trial [10].

An NA-deficient influenza vector with RBD embedded into NA was developed by Loes et al. [11]. In this construct, the RBD is targeted to membrane expression, and mouse studies confirmed that such vaccines are immunogenic in terms of the induction of RBD-specific antibodies. Later, the same RBD cassette was inserted into the NS gene of the M2-deficient H3N2 influenza virus vector [12], also leading to high level of membrane expression of the RBD. Another interesting influenza vector design is developed by a group of scientists from Nanjing Agricultural University [13]. In this development, RBD is incorporated into the virus membrane, whereas the HA and NA proteins of the virus are substituted by the influenza C hemagglutinin-esterase-fusion glycoprotein. The incorporation of the RBD into the virus particle provided immunogenicity against SARS-CoV-2 but the potential of this vaccine as an influenza vaccine is controversial due to the absence of major antigens that are necessary for protection against influenza A infection. Another design exploiting HA UTRs and transmembrane domain was developed in Thailand [14]. This virus is characterized by a single-cycle replication, due to the absence of the HA sequence in the vector genome, and hence the vaccine requires HA-expressing cell line for production. An additional strategy of RBD incorporation in influenza particles is described by Chaparian et al. [15], where the RBD-encoding fragment fused with the transmembrane domain from the influenza NA is inserted into the HA gene through the P2A self-cleavage site.

Despite the wide list of bivalent influenza and COVID-19 vaccines under development, there are no such vaccines licensed for mass immunization yet, and the development of new candidate vaccines based on well-characterized backbones seems to be relevant. In this work, we used a licensed live attenuated influenza vaccine (LAIV) backbone virus [16] as a vector to incorporate SARS-CoV-2 RBD fragments. Several types of RBD-encoding cassettes were inserted into the HA or NS genes of influenza virus. Nine variants of recombinant vaccine candidate strains were rescued and assessed in in vitro experiments, as well as in animal models. The most promising vaccine candidate was investigated in an experiment on golden Syrian hamsters, challenged by SARS-CoV-2 and influenza viruses.

## Materials and methods

### Viruses, cells and proteins

#### Cells

Two African green monkey cell lines (Vero ATCC CCL-81 and Vero E6 ATCC C1008), and canine kidney cell culture (MDCK, ATCC CCL-34) were maintained according standard protocol in Dulbecco’s Modified Eagle Medium (DMEM) supplemented with 10% fetal bovine serum (FBS) and 1×antibiotic-antimycotic solution (all from Capricorn, Germany) at 37°C and 5% CO_2_.

#### Viruses

A previously rescued live attenuated influenza vaccine (LAIV) virus served as a viral vector for generation of recombinant influenza viruses expressing immunogenic fragments of SARS-CoV-2 [17]. This virus carries hemagglutinin (HA) and neuraminidase (NA) genes of A/17/Anhui/2013/61 (A/Anhui/1/2013-based LAIV strain) and the remaining six genes from A/Leningrad/134/17/57 (H2N2), a master donor virus for LAIV. An H7N9 LAIV virus expressing truncated NS1 protein was used here as an additional vector control; generation and main characteristics of this vaccine virus were previously reported [17].

A reassortant influenza virus PR8-IDCDC-RG32A (Sh/PR8) carrying HA and NA genes of A/Shanghai/2/2013 (H7N9) virus and the remaining genes of A/PR/8/34 (H1N1) strain was obtained from the Centers for Disease Control and Prevention (CDC, Atlanta, GA, USA).

Influenza viruses were propagated in 10–11-day-old embryonated chicken eggs at 33°C (for attenuated viruses) or 37°C (for Sh/PR8 virus), clarified by low-speed centrifugation and stored at -70°C. For virus concentration, the clarified allantoic fluid was subjected to ultracentrifugation on a 30%/60% sucrose gradient, as previously described [18].

The SARS-CoV-2 virus, HCoV-19/Russia/StPetersburg-3524/2020 (Wuhan lineage, D614G, GISAID ID EPI_ISL_415710) was obtained from the Smorodintsev Research Institute of Influenza (Saint Petersburg, Russia). This virus was cultured in Vero (ATCC CCL-81) cells grown in DMEM supplemented with 2% FBS (DMEM/2% FBS) with addition of 1× antibiotic-antimycotic solution (Capricorn, Germany) and 10 mM HEPES at 37°C and 5% CO_2_. Cell supernatants were harvested 72-96 h after inoculation and aliquoted into single-use stock vials after centrifugation at 3500 rpm for 15 min. The sucrose-gradient purified viruses were obtained using a previously described method of the high-speed centrifugation in sucrose gradient [18].

#### Proteins and peptides

The recombinant RBD of spike protein of SARS-CoV-2 (Wuhan lineage) was stably expressed in HEK293 cells and provided by JSC «BIOCAD» [19]. The recombinant full-length HA protein of A/Shanghai/2/2013 (H7N9) virus was kindly provided by Professor Florian Krammer (Mount Sinai School of Medicine, New York, USA).

For cell stimulation in ELISPOT analyses, the pools of SARS-CoV-2 peptides – PepTivator® SARS-CoV-2 Prot_S, Prot_N (Miltenyi Biotec, Germany) – were used. All proteins were stored at −70°C in aliquots.

### Design of the chimeric influenza genes expressing RBD

The modified HA gene of A/17/Anhui/2013/61 (A/Anhui/1/2013-based LAIV strain) was designed based on previously studied experimental RSV vaccine [20]. The specific part of the RBD was selected based on immunogenicity and conformation stability data. The RBD-coding fragment was inserted into the plasmid encoding full-length influenza HA, immediately after the end of the signal peptide-encoding sequence. In the final construct, the RBD fragment was fused to the N-end of the HA1 subunit through the GGGGSGGGGS flexible linker.

In the modified versions of the A/Leningrad/134/17/57 NS gene, the ORF of the NS1 protein was truncated to 126 amino acid residues, as described previously [21, 22]. The SARS-CoV-2 genetic material was inserted after the 126^th^ codon of NS1 in several modifications: (1) after the P2A-encoding sequence, (2) connected to the NS1 through the GGGGS linker, and (3) after the Stop-Start pentanucleotide of influenza B virus TAATG [23]. The residual part of the NS1 gene was truncated.

The genetic material was inserted into previously prepared pCIPolISapIT plasmids with the A/Anhui/1/2013 HA or the A/Leningrad/134/17/57 NS genes using overlap PCR or Golden Gate cloning with BsmBI endonuclease (New England Biolabs, Massachusetts, USA).

### Rescue of recombinant influenza viruses

The rescue of recombinant viruses was performed with the same procedure as described previously [21]. In brief, purified plasmids encoding all influenza genes (PB2, PB1, PA, NP, M of A/Leningrad/134/17/57, the NA of A/Anhui/1/2013, modified or non-modified NS from A/Leningrad/134/17/57, and modified or non-modified HA from A/Anhui/1/2013) were mixed (2 μg of each plasmid) for transfection of Vero cells. Electroporation of Vero cells was performed with Neon Transfection system (Thermo Fisher Scientific, Massachusetts, USA) according to the manufacturer’s manual. After the electroporation procedure, the cells were incubated for 6 hours at 37°C, 5% CO2 for attachment, and then the medium was replaced with OptiPro SFM with 1x GlutaMax (Gibco), 1x antibiotic-antimycotic and 2.5 μg/mL trypsin (Sigma, Burlington, MA, USA). After this step, the cells were incubated at 33°C, 5% CO2 for 72 hours. Then, the cells were detached from dishes with cell scraper and resuspended in the cell culture medium, followed by inoculation of 10-11-days old developing chicken embryos. After incubation at 33°C for 72 hours, the virus was detected in allantoic fluid by standard hemagglutination test with 0.5% chicken red blood cells. The genetic identity of the recombinant virus genes was confirmed by Sanger sequencing. For this, RNA was extracted from virus-containing allantoic fluid using RNA extraction kit (Biolabmix, Novosibirsk, Russia), followed by PCR with reverse transcription using One-Step RT-PCR kit (Biolabmix, Novosibirsk, Russia) and specific primer sets. cDNA was extracted from agarose gel and subjected to sequencing reaction using BigDye™ Terminator v3.1 Cycle Sequencing Kit (Thermofisher Scientific, Massachusetts, USA). The reaction was further analyzed with 3130xl Genetic Analyzer (Applied Biosystems, USA). In case of mutations additional passage variants were sequenced.

### In vitro studies of the recombinant influenza viruses

#### Replication in eggs and genetic stability

The infectious virus titers were assessed in 10-11 days old developing chicken embryos at 33°C for 72h, and the titers were calculated according to the Reed and Muench method [24] and expressed as log_10_EID_50_/mL. The genetic stability of the recombinant influenza viruses was assessed after 3, 5 and 10 sequential passages in eggs. The identity of the chimeric influenza virus genes containing SARS-CoV-2 inserts was evaluated by Sanger sequencing.

#### Expression of RBD protein in MDCK cells infected with recombinant influenza viruses

The RBD expression in MDCK cells infected with the recombinant viruses was assessed by sandwich ELISA of lysed cells with anti-RBD antibodies. MDCK monolayers were infected with test viruses at 0.002 MOI and incubated at 33°C, 5% CO2. At 60 hours post infection, cells were lysed with lysis buffer (250 mM sucrose, 50 mM Tris-HCl, 25 mM NaCl, 2 mM EDTA, 0.1% Triton X-100, 1 µg/mL trypsin inhibitor, 1 mM PMSF) on ice for 5 minutes with periodic mixing. Cell debris was pelleted by centrifugation at 13000 g and 4°C for 10 minutes. The supernatant was tested by ELISA in high-sorbent 96-well plates coated with rabbit polyclonal anti-RBD antibody (BIOCAD, Russia), 100 ng/well. Fourfold dilutions (from 20 µg/ml to 1.2 ng/mL) of a recombinant RBD protein expressed in mammalian cells [19] was used for standard curve generation. After blocking with 5% skim milk, the cell lysate supernatant was added in triplicates and incubated for 1h, followed by 2× washing with PBST. Then, a mouse monoclonal anti-RBD antibody (kindly provided by Dr Alexey Sokolov, FSBSI “IEM”, St. Petersburg) was added to the plates at a concentration of 1 mg/mL. After 1-hour incubation and washing, HRP-conjugated goat anti-mouse antibody (BioRad, USA) was added to the wells and incubated for 1 hour at 37°C. Plates were washed with PBS-T and the color was developed with 1-Step TMB Substrate Solution (HEMA, Russia). The reaction was stopped with 1 M H_2_SO_4_, and the resulting absorbance was measured at wavelength 450 nm (OD_450_) using xMark Microplate Spectrophotometer (BioRad, USA). The RBD expression level was calculated by approximation of the mean OD_450_ value to the standard curve generated from the recombinant RBD protein.

#### SDS-PAGE and Western blot analysis

The SDS-PAGE and Western blot analysies were performed with sucrose-purified viruses taken at an equal protein concentration of 0.5 mg/mL of each. The discontinuous SDS-PAGE was performed under non-reducing conditions according to the previously described method [25] in 5% stacking and 10% resulting polyacrylamide gels loaded with the samples, which were prepared by mixing of sucrose gradient-purified viruses with a loading Laemmli buffer. The resulting gels were blotted as previously described [26] on the nitrocellulose membranes with a pore diameter of 0.45 μm. The membranes, blocked with 5% skimmed milk on PBST for 1 h at 37°C, were overnight-incubated with rabbit polyclonal anti-RBD antibodies (5 µg/ml in blocking buffer; Bio-Rad, USA) or mouse polyclonal anti-H7 serum raised to the recombinant H7 HA protein (1:200) at 4°C. The next day, the membranes were washed three times and immunodetected with anti-mouse or anti-rabbit secondary antibodies conjugated with horseradish peroxidase (1:3000 in PBS-T, Bio-Rad, USA) for 1 h at 37°C. Finally, 0.05% solution of diaminobenzidine (Sigma, USA) in PBS containing 1% hydrogen peroxide was used to stain the treated membranes.

### Animal studies

Animal experiments were performed according to the Directive 2010/63/EU of the European parliament and of the council of September 22, 2010, on the protection of animals used for scientific purposes [27]. Animal study designs were approved by local ethics committees (local ethics committee of FSBSI “IEM” protocol 1/22 on 18.02.2022).

All animals were quarantined before the start of the studies, and were examined for the presence of antibodies to influenza and coronavirus before the start of the experiment.

#### Replication and immunogenicity in BALB/c mice

Female BALB/c mice were purchased from the Stolbovaya farm (Moskow region, Russia). Mice were immunized intranasally with 10^6^ EID_50_ of each experimental vaccine strain, in a volume of 50 μL. Viral titers in respiratory tissues were assessed at 3 dpi. For this, mice were euthanized with isoflurane, and the nasal turbinates and lungs were aseptically collected and stored at -70°C. Organs were homogenized using a small bead mill (TissueLyser LT, QIAGEN, Germany) in 1 mL of sterile PBS. Titers were determined in eggs as described above and expressed as log_10_EID_50_/mL.

For immunogenicity studies, animals were immunized twice with the same vaccine dose, at a 3-week interval. Serum samples and spleens were collected 21 days after the second dose. Immune responses to influenza were studied in enzyme-linked immunosorbent assay (ELISA) against a sucrose-gradient purified whole influenza virus; antibody responses to the SARS-CoV-2 were assessed in ELISA against RBD recombinant protein and, as well as in a microneutralization (MN) test of live SARS-CoV-2.

#### Replication, immunogenicity and protective activity in Syrian hamsters

Six- to eight-week-old female Syrian hamsters were intranasally immunized with 5×10^6^ EID_50_ of the studied influenza viruses, or with PBS. To determine the ability of the vaccine prototypes to replicate in the upper (URT) and lower (LRT) respiratory tracts of immunized hamsters, nasal and lung tissue samples were collected on day 3 after the first vaccine dose (4 animals from each group). Viral titers in tissue homogenates were determined by titration in eggs as described above. To study antibody responses to influenza and SARS-CoV-2, animals were immunized twice with a 3-week interval with the 5×10^6^ EID_50_ and the sera were collected at day 21 after the second immunization. To assess protective potential against influenza virus and SARS-CoV-2, groups of hamsters immunized twice with the recombinant virus, as well as with the H7N9 LAIV vector, and mock-immunized animals were i.n. challenged 3 weeks after the second dose with Sh/PR8 influenza virus at a dose of 10^6^ EID_50_ or with SARS-CoV-2 Wuhan (D614G) at a dose of 10^5^ TCID_50_. Influenza virus-infected animals were euthanized 4 days post challenge and viral titers in tissue homogenized were determined by titration in eggs as described above. SARS-CoV-2-challenged hamsters were monitored for weight loss and clinical symptoms for 5 days after infection. The scoring of behavior and clinical signs was assessed as the sum of declining of following signs: behavior in the cage (normal=0; depressed=1); behavior in the open area (normal=0; sluggish=1); reaction to taking in hands (normal=0; sluggish=1); fur condition (normal=0; lack of grooming=1); interest to food (normal=0, decreased=1). On day 5 after challenge, animals were euthanized and lungs, nasal turbinates and spleens were collected for virological, immunological and/or histopathological evaluations. Viral titers in the URT and LRT were determined by titration tissue homogenates in Vero cells and expressed as lg TCID_50_/gram tissue. Spleens were used to measure recall T-cell immunity to SARS-CoV-2 antigens in the ELISPOT assay.

### Assessment of immune responses

#### ELISA

The levels of serum antigen-specific antibodies were assessed by ELISA. Briefly, 96-well high-binding polystyrene plates (Thermo Scientific, USA) were coated with sucrose-gradient purified H7N9 LAIV virus (16 HA units per well) or with recombinant RBD protein (100 ng per well) in carbonate-bicarbonate buffer (pH 7.4) for 20 hours at +4°C. The coated plates were 3 times washed with PBS-T, and 2-fold serum dilutions were added in duplicates for 1 h at 37°C. After intensive washing with PBS-T a solution of secondary anti-mouse (Bio-Rad, USA) or anti-hamster (Thermo Scientific, USA) IgG HRP-conjugated antibodies were added to the plates and incubated for 1 h at 37°C. Thoroughly washed plates were finally developed with a 1-Step TMB Substrate Solution (HEMA, Russia). The reaction was stopped with 1 M H_2_SO_4_, and the resulting absorbance was measured at 450 nm (OD_450_) using xMark Microplate Spectrophotometer (BioRad, USA). Antibody titers were determined as the last serum dilution with the OD_450_ value exceeding twice the mean OD_450_ values of the control wells (no serum added).

#### Microneutralization

The microneutralization test was performed as previously described [18]. In brief, 300 TCID_50_ of SARS-CoV-2 virus were mixed with 2-fold dilutions of serum samples and incubated for 1 h, followed by mixture transfer to 96-well plates with confluent monolayers of Vero cells. After 1 h of incubation at 37°C, 5% CO_2_, the inoculum was removed, culture medium containing corresponding serum dilutions was added to appropriate wells, and the plates were incubated for 48 h at 37°C, 5% CO_2._ After incubation, medium was removed and cells were fixed with 2% formaldehyde in PBS solution, and virus replication was detected using ELISA with rabbit anti-RBD antibodies (BIOCAD, Russia) and secondary HRP-conjugated anti-rabbit IgG antibodies (BioRad, USA). The color was developed with a 1-Step TMB Substrate Solution (HEMA, Russia) and optical density was measured at wavelength 450 nm using xMark Microplate Spectrophotometer. The 50% inhibitory concentration (IC_50_) was calculated with a four-parametric nonlinear regression method.

#### ELISPOT

The IFN-γ response of isolated splenocytes and lung cells of Syrian hamsters on the 5th day after challenge was measured using an IFN-γ ELISPOT Plus kit (Mabtech, Sweden) according to the manufacturer's protocol and as described in [21]. Briefly, a pre-coated with monoclonal antibody ELISPOT plate was washed 4 times with sterile PBS (200 µl/well) and then incubated with the CR-10 medium (RPMI supplemented with 10% FBS, 5 mM HEPES, 1× antibiotic-antimycotic, and 50 μM β-mercaptoethanol) for 30 minutes at room temperature. Then, CR-10 media was removed and “cells + stimuli” mixtures were added to each well, followed by 18h incubation at 37°C, 5% CO2. 500,000 cells were stimulated either with 0.1 MOI of purified SARS-CoV-2 or with 1 MOI of purified influenza virus, or with PepTivator S + N mixture (30 pmol per peptide) (Miltenyi Biotec Bergisch Gladbach, Germany). The detection of spots was performed according to the manufacturer’s protocol using detection antibody and substrate solution. Color development was stopped by extensively washing in tap water. Before counting, the plate was left to dry overnight, then spots were counted in an AID vSpot Spectrum reader (Advanced Imaging Devices, Germany).

### Histopathological analyses

Lung tissues of Syrian hamsters immunized with tested vaccines or mock-immunized (PBS) and challenged with SARS-CoV-2 were subjected to histopathological evaluation using previously described methodology [21]. Briefly, the lungs (*n*=4 per group) were fixed in 10% neutral buffered formalin for at least 48 hours. The tissues were embedded in paraffin by Tissue-Tek VP1 station (Sakura, Japan), histological sections (l-3 μm) were prepared and stained with hematoxylin and eosin. Morphometric measurements included semiquantitative assessment of airway damage (comprised of % airway affected, airway severity and bronchiolar epithelial hyperplasia), lung/alveolar damage (comprising of % alveoli affected, alveolar severity and type II pneumocyte hyperplasia), and vascular damage (comprising of % vessels affected, vascular/perivascular lesions and necrotizing vasculitis/thrombi) according to [28]. The detailed information of the scoring criteria was published earlier [21].

### Statistical analyses

The results were analyzed using GraphPad Prism 7.0 software. The parameters of distribution were assessed with Shapiro-Wilk normality test. For group comparisons, one-way ANOVA with post-hoc Tukey’s test was used, or the Kruskal-Wallis test with post-hoc Dunn’s test. The differences were considered significant at *p* < 0.05.

## Results

### Generation of chimeric influenza viruses

We used two strategies of influenza virus modification to deliver SARS-CoV-2 antigens to target cells. The first one involves modification of the HA gene of influenza virus, which includes incorporation of the antigenic fragment of SARS-CoV-2 into the influenza virion as a structural part of the HA protein. In the second case, we modified the NS gene of the LAIV viral vector to ensure independent processing of influenza and SARS-CoV-2 antigens in infected cells.

#### Generation of recombinant influenza viruses with modified HA genes

**Fig. 1:**
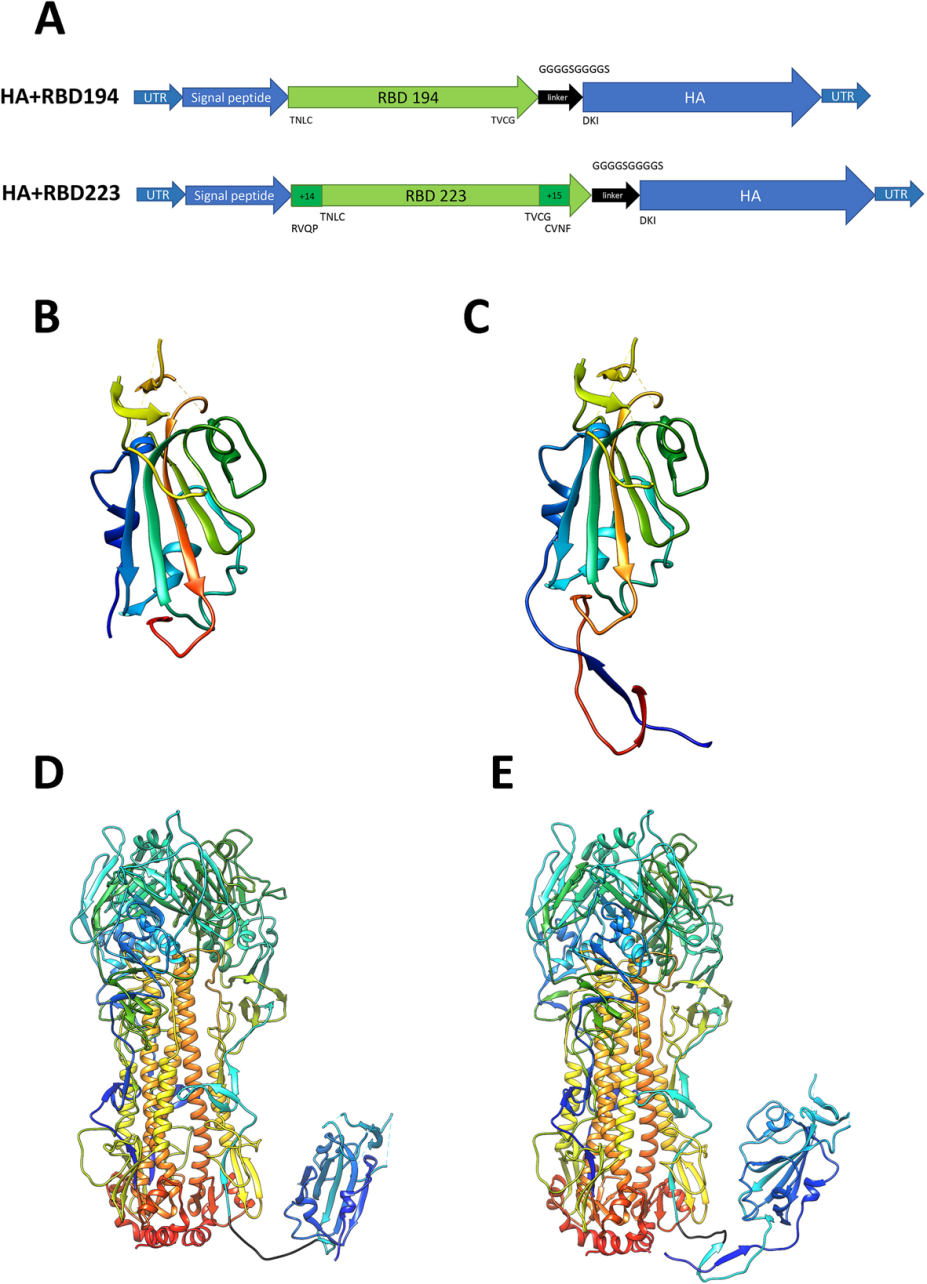
Schematic representation of the chimeric HA constructions. **A** schematic representation of the chimeric HA genes encoding RBD fragments of the SARS-CoV-2 spike protein. **B**-**E** schematic visualization of SARS-CoV-2 RBD-based cassettes inserted into HA. **B** RBD 194 cassette (based on PDB 6vxx); C RBD 223 cassette (based on PDB 6vxx); **D** H7 HA trimer with RBD 194 cassette connected to one of the three HA monomers. The linker is colored in black; **E** H7 HA trimer with the RBD223 cassette connected to one of the three HA monomers. The linker is colored in black. Figures were prepared using UCSF Chimera 1.11.2 [34]

We designed SARS-CoV-2 RBD-based cassettes for incorporation into influenza HA molecules because this strategy was successfully used in our previous studies [20, 29]. We inserted the RBD-encoding fragment into the H7N9 influenza virus HA gene between the signal peptide-encoding sequence and the HA1 subunit of the molecule using the GGGGSGGGGS flexible linker. It was shown in previous experiments that cassettes in such constructs are expressed as a part of HA protein and exposed at the surface of the virion [20, 30]. According to our previous studies, the size of the cassette may have a significant impact on the virus growth characteristics and immunogenicity [31]. Therefore, we designed two variants of the SARS-CoV-2 RBD-based immunogenic cassettes of different lengths. The first variant, HA+RBD 194, contained the insertion of the SARS-CoV-2 spike protein’s 333-526 amino acid residues, as described in [32]. Another construct was designed based on the full-length RBD protein of the Wuhan strain which comprises 223 amino acid residues (319-541) of the spike protein [33] (Fig. 1).

We rescued two recombinant influenza viruses expressing chimeric HA proteins as shown in Fig. 1, and carrying intact N9 gene, as well as six remaining genes of A/Leningrad/17 LAIV master donor virus. The chimeric viruses encoding the inserts RBD 194 and RBD 223 were named FluCoVac-19 and FluCoVac-20, respectively (Table 1).

**Table 1:** Recombinant viruses with SARS-CoV-2 RDB fragments incorporated into HA molecule

**Virus name**	**Type of insert**	**Viral titer in eggs, lgEID**_**50**_**/mL**	**Genetic stability of the insert**^**a**^
LAIV	-	9.8	-
FluCoVac-19	RBD 194	8.4*	A single mutation in the (G_4_S)_2_ linker
FluCoVac-20	RBD 223	8.3*	A single mutation in the (G_4_S)_2_ linker

^a^genetic stability was assessed after 10 passages in eggs

^*^Significantly reduced titer (*p*<0.05) compared to the LAIV vector control virus

The rescued LAIV-RBD viruses were amplified in eggs, and their titers ranged from 8.3 to 8.4 lgEID_50_/mL (Table 1). Although they were significantly lower than that of the H7N9 LAIV vector, such infectious activity of the recombinant strains is suitable for further manufacturing processes. Ten sequential passages in eggs of the rescued viruses revealed high genetic stability of the RBD 194 (FluCoVac-19) and RBD 223 (FluCoVac-20) inserts, since only a single substitution was found in each virus, both times within the flexible linker: GGGGSG**R**GGS in FluCoVac-19 virus and GGGG**N**GGGGS in FluCoVac-20 variant. It is very unlikely that these minor changes will affect the antigenicity of the chimeric HA molecule, since the RBD sequence remained unchanged.

#### Generation of recombinant influenza viruses with modified NS genes

We also designed a panel of RBD-based constructs for their expression in the target cells as part of the modified NS gene of the LAIV virus. We previously developed recombinant LAIV strains with modified NSs that successfully stimulated T-cell immunity to other respiratory pathogens, such as RSV [35] and human adenovirus [22]. Here, we explored different variants of influenza NS gene modifications, targeting strategies and transgene cassette processing pathways that were found to be prospective for other recombinant vaccines.

**Fig. 2:**
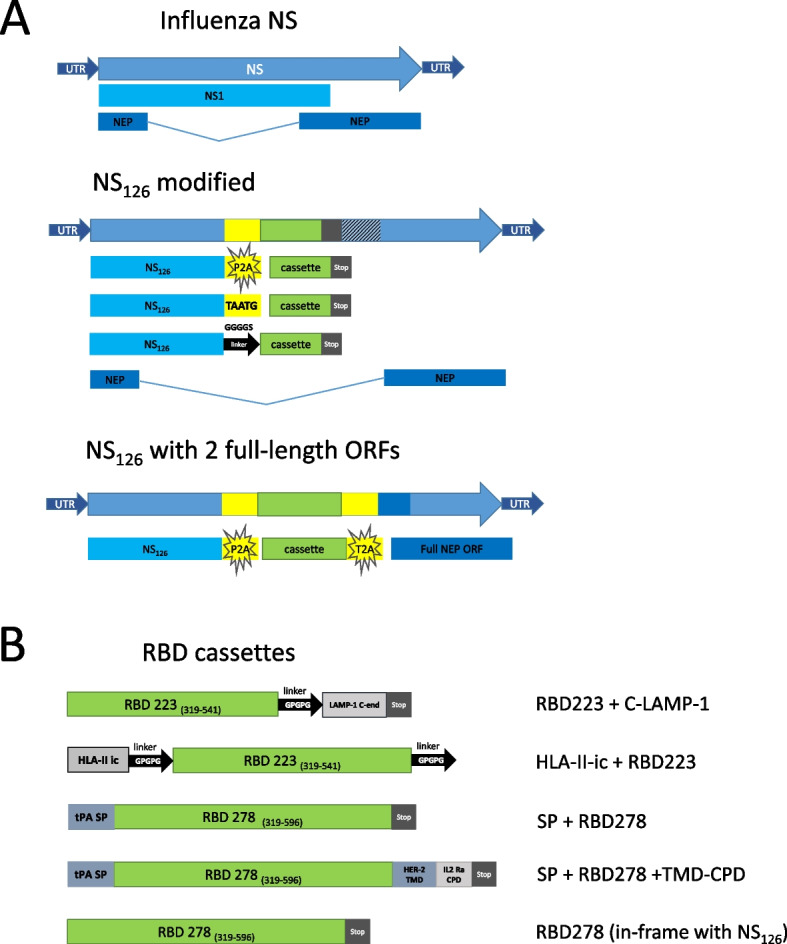
Schematic representation of the chimeric NS1 genes encoding RBD fragments of the SARS-CoV-2 spike protein, along with different targeting signals. **A** Types of modifications of the influenza NS1 gene; **B** Types of RBD cassettes inserted into NS1 ORF. SP: signal peptide. TMD: transmembrane domain, erbB-2 (HER-2). CPD: cytoplasmic domain (alpha-subunit of the IL-2 receptor CPD)

We tested two modifications of the NS gene sequence. In the first type, the NS1 coding region was truncated up to 126 residues, followed by the linker and the RBD-based insert. The noncoding fragment of the NS1 ORF was removed from the sequence, except for the regions necessary for splicing for NEP. In another type of construct, we removed all noncoding sequences of NS1 and added necessary sequence after the cassette insertion for full-length NEP ORF recovery, through the T2A self-cleavage site (Fig. 2A). This strategy was previously studied by DiPiazza et al. [36]. In addition to the intact RBD fragment, we designed four variants of the RBD inserts with different targeting sequences that were supposed to enhance the humoral immune response to the transgene.

Two strategies were used for SARS-CoV-2 cassette targeting to lysosomal compartment. A human LAMP-1 transmembrane C-end peptide sequence (35 amino acid residues) was added to the C-end of the RBD cassette (Fig. 2B). Early studies on DNA antiviral vaccines demonstrated that the addition of this peptide to an antigen leads to enhanced immunogenicity [37–39]. The mechanism is based on enhanced antigen presentation through the MHC class II pathway. In the second variant, the HLA-II invariant chain with transmembrane anchor domain was added to the N-end of the RBD cassette to serve as a signal of lysosomal targeting [40]. The role of the HLA-II invariant chain in the regulation of Th immune responses [41] suggested using it as a part of the transgene to modulate immune responses to DNA vaccination. In the mouse model, incorporation of CD4+ peptide into the HLA-II invariant chain led to high level of Th immune response after the peptide stimulation [42].

The other two designs included the tissue plasminogen activator (tPA) signal peptide at the N-terminus of the RBD cassette, and one of them also included the HER-2 transmembrane domain (TMD) and the IL2Ra cytoplasmic domain (CPD) fused to the C-end of RBD cassette (Fig. 2B). The tPA signal peptide is a signal sequence often used to transport the attached proteins towards the secretion pathway in mammalian cells. This signal peptide was used in several studies and its addition enhanced the immunogenicity of different constructs (MVA-based vaccine against tuberculosis, DNA vaccine with HIV-1 antigen, DNA vaccine against influenza) [43–45]. The idea is based on direct presentation of the target protein to immune cells in the bloodstream. In our case, the RBD structure was further stabilized by additional 55 residues of the spike protein and a single mutation (see below, [19]).

The usage of membrane targeting is a common strategy for enveloped viral vector presentation of foreign antigens. This strategy is widely used in recombinant baculoviruses [46]. The membrane targeting of the antigen in vector vaccines can improve immunogenicity even if the antigen is not originally membrane-bound [47]. The effect of different cytoplasmic domains on the protein’s effective surface presentation and overall vaccine potential was also studied [48]. Here, we tested the erbB-2 (HER-2) transmembrane domain fused with the alpha-subunit of the IL-2 receptor (IL2Ra) cytoplasmic domain, which was described earlier [49] (Fig. 2B). This combination was proven to be useful in previous experiments on cell lines development, e.g., for overexpression of human Fc receptors on the surface of CHO and HEK293 cells (data not shown). Also, there are no tyrosine residues in IL2Ra cytoplasmic domain, and therefore there is no chance of interference with complex signaling pathways in target cells, unlike the biologically active HER2 cytoplasmic domain.

Therefore, we tested several different approaches to trigger and enhance the immune response toward the key receptor-binding domain of the SARS-CoV-2 spike protein, along with the response to the influenza virus.

We also tested three ways to insert the RBD-based cassettes into truncated NS1 ORF (Fig. 2A): (i) using the P2A self-cleavage site previously described for designing T-cell-based vaccines and which ensured independent processing of influenza virus antigens and the inserted transgene [21, 35]; (ii) via the pentanucleotide Stop-Start codon (TAATG) used by influenza B virus to terminate and reinitiate translation, which was tested earlier to express reporter GFP or functional IL-2 [50] or RSV epitopes [51, 52] from the truncated NS1 ORF; and (iii) by fusing the NS_126_ protein with the RBD fragment through a flexible linker, so that the both proteins are expressed without disintegration. This strategy was shown to be promising in several studies of influenza viruses as viral vectors [53–55].

Of note, most of our RBD inserts were designed to have a prolonged region of the spike protein, comprising of residues 319-596 (278 amino acids), since this modification was proven to be highly immunogenic when expressed by an adeno-associated virus (AAV) vector [19].

Overall, we rescued seven recombinant LAIV viruses expressing various RBD-based cassettes from the modified NS1 protein ORF, as listed in Table 2. FluCoVac-35, FluCoVac-41, FluCoVac-59 and FluCoVac-72 encoded different RBD cassettes following the P2A autocleavage site, whereas FluCoVac-78 and FluCoVac-79 were connected to the NS1 protein fragment via the Stop-Start pentanucleotide. The last variant, FluCoVac-83, encoded the RBD antigen in-frame with the NS1 and was separated from the influenza protein by the flexible linker.

**Table 2:** Recombinant viruses with SARS-CoV-2 RDB fragments incorporated into NS1 ORF

**Virus name**	**Type of insert**	**Type of linker**	**Size of the insert, nt**	**Viral titer in eggs, lgEID**_**50**_**/mL**	**Genetic stability of the insert**^**a**^
LAIV-NS_126_	-	-		7.9	-
FluCoVac-35	RBD223+cLAMP	P2A	864	6.8	No mutations found
FluCoVac-41	HLA-II-ic-RBD223	P2A-T2A	996	7.9	Unstable
FluCoVac-59	SP-RBD278	P2A	978	7.3	No mutations found
FluCoVac-72	SP-RBD278-TMD-CPD	P2A	1086	8.5	No mutations found
FluCoVac-78	SP-RBD278	Stop-Start	913	6.7	No mutations found
FluCoVac-79	SP-RBD278-TMD-CPD	Stop-Start	1021	5.7*	No mutations found
FluCoVac-83	RBD278	GGGGS	861	8.3	No mutations found

^a^genetic stability was assessed after 10 passages in eggs

^*^Significantly reduced titer (*p*<0.05) compared to the LAIV vector control virus

The replicative activity of the recombinant viruses in chicken embryos was compared to that of the modified H7N9 LAIV virus encoding truncated to 126 residues NS1 protein, which was characterized earlier [17]. Most of the chimeric influenza viruses replicated efficiently in eggs, except FluCoVac-79 variant (Table 2). It is likely that this combination of the RBD cassette and the TAATG linking region interfered with the infectious activity of the chimeric virus. Interestingly, the same RBD cassette with SP, TMD and CPD inserted via the P2A self-cleavage site had no negative effect on the growth properties of the recombinant virus; in fact, this variant, FluCoVac-72, replicated better in eggs than the control LAIV NS_126_ vector virus (Table 2).

Serial passaging of the rescued LAIV/RBD variants revealed high level of genetic stability of all but one recombinant virus (Table 2). Strikingly, the only variant that encoded the full-length NEP following the RBD cassette and the T2A autocleavage site (FluCoVac-41) was unstable and the insert was not detected in the virus after six passages in eggs. However, more research is needed to elucidate the exact genetic mechanisms underlying this phenomenon.

### Expression of the RBD protein by the chimeric LAIVs

#### Expression in MDCK cells

**Fig. 3:**
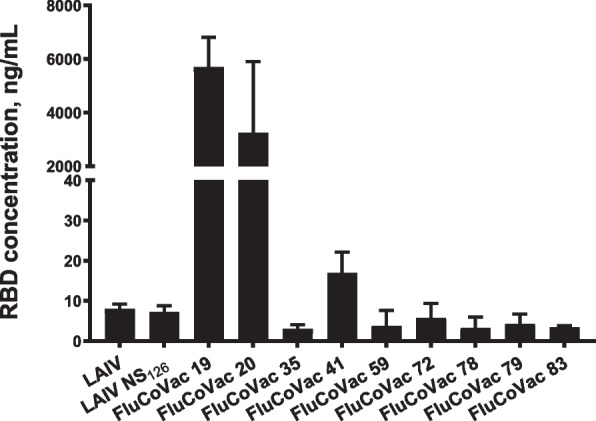
Expression of RBD protein by recombinant LAIV/RBD viruses in infected MDCK cells. The cells were infected with each virus in triplicates and the concentration of RBD in cell lysates was measured 60 hpi by sandwich ELISA

The expression of correctly folded RBD protein in MDCK cells infected with experimental vaccine strains was evaluated by sandwich ELISA of cell lysates. The productive infection of the cell with each recombinant virus was confirmed by hemagglutination assay of cell supernatants, as well by the detection of cytopathic effect in each virus-infected well. Unexpectedly, the expression of high levels of RBD protein was detected only in cells infected with two variants with RBD insertions into the influenza HA molecule – FluCoVac-19 and FluCoVac-20 (Fig. 3). No significant expression of the RBD was detected in MDCK cells infected with any of the recombinant viruses with insertions into the NS1 ORF, indicating that synthesis of the RBD protein from the NS1 open reading frame does not result in proper folding of the target antigen within the infected cell (Fig. 3).

#### Western blot

**Fig. 4:**
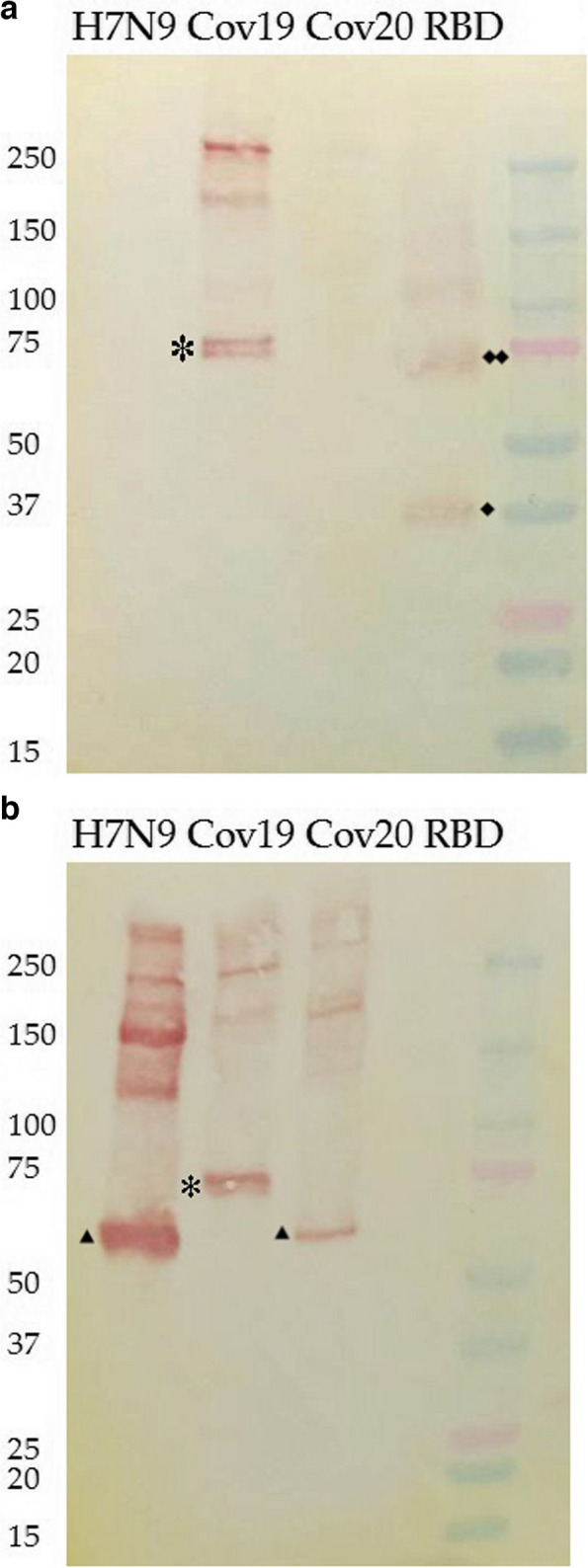
Western blot analysis of sucrose gradient-purified influenza viruses and a recombinant RBD protein using: **A** anti-RBD rabbit polyclonal antibody (*) – influenza HA monomer with RBD insertion; (♦) – monomeric recombinant RBD (♦♦) – dimeric form of RBD; the higher bands are oligomers of these forms; **B** anti-H7 HA mouse hyperimmune sera. (*) – influenza H7 HA monomer with RBD insertion is higher than H7 HAs without insertions (triangle); Cov19: FluCoVac-19. Cov20: FluCoVac-20. The H7N9 LAIV vector (H7N9) and recombinant RBD protein (RBD) were used as control antigens in this assay

Since RBD fragments inserted into HA molecule of influenza virus are supposed to be exposed on the surface of the virion, we conducted Western blot analyses of the sucrose gradient-purified viruses FluCoVac-19 and FluCoVac-20, using a polyclonal anti-RBD rabbit antibody and a mouse hyperimmune sera raised to the recombinant H7 HA protein expressed in insect cells. The H7N9 LAIV vector, as well as recombinant RBD protein, were used as control antigens in this assay. As shown in Fig. 4A, three apparent bands reacting with anti-RBD antibodies were observed in the FluCoVac-19 virus, suggesting the presence of RBD antigen in complex with the monomeric, dimeric and trimeric influenza HA molecules. The recombinant RBD protein used as a positive control in this study was also detected by anti-RBD antibodies in monomeric and multimeric forms, each monomer with expected size about 35 kDA (rhombus at Fig. 4A). Unexpectedly, no anti-RBD antibody binding was detected in the case of the FluCoVac-20 variant (Fig. 4A), whereas clear RBD expression was noted when MDCK cells were infected with this virus (Fig. 3). The absence of the RBD fragment within the HA molecule of FluCoVac-20 was confirmed by Western blot with anti-H7 antibody: the HA bands in various forms in this variant were identical to the H7N9 LAIV control virus, whereas corresponding bands of the FluCoVac-19 virus appeared at higher molecular weight, confirming the presence of an additional fragment within this antigen (Fig. 4B). Since FluCoVac-20 encoded the RBD fragment within the chimeric HA gene, which was confirmed by Sanger sequencing of the purified virus material, and expressed significant quantities of RBD within infected MDCK cells, most likely that the RBD fragment in this virus is subjected to proteolytic cleavage post-translationally and is not exposed on the surface of the virion.

### Replication and immunogenicity in BALB/c mice

Despite the lack of RBD protein expression in some of the rescued recombinant LAIV/RBD viruses, all variants were assessed in a mouse model to determine their replicative activity in the respiratory tract and their ability to induce antibody responses to the whole influenza virus, as well as to the target RBD antigen. We hypothesized that the use of a monoclonal antibody in an ELISA expression assay could give a false negative result in the case of incorrect folding of the only epitope to which the antibody is specific, but that an immune response to other epitopes could form correctly.

**Fig. 5:**
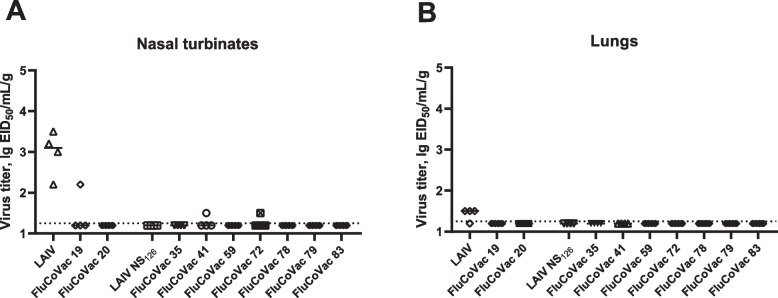
Replication of experimental viruses in BALB/c mouse nasal turbinates (**A**) and lung tissue (**B**). BALB/c mice were immunized with experimental vaccine strains at a dose of 10^6^ EID_50_ and tissues were collected on day 3 post immunization. Influenza viral titers were determined in eggs

Groups of mice were i.n. inoculated with 10^6^ EID_50_ of each virus, and the lungs and nasal turbinates were collected on day 3 post infection. As shown on Fig. 5, very weak replication was detected in all recombinant viruses. The FluCoVac-19 and FluCoVac-20 variants were compared to the classical LAIV virus, and the difference in the replicative activity between chimeric and control viruses suggests that the foreign insert could have interfered with the ability of the LAIV virus to replicate in the mouse URT. For the NS1-modified recombinant viruses, the absence of infectious virus in the mouse respiratory tract is in line with findings that the LAIVs encoding truncated NS1 protein had restricted ability to infect mice [17]. Therefore, the effect of foreign insertions within the NS1 ORF on the infectivity of the virus in mice could not be elucidated.

**Fig. 6:**
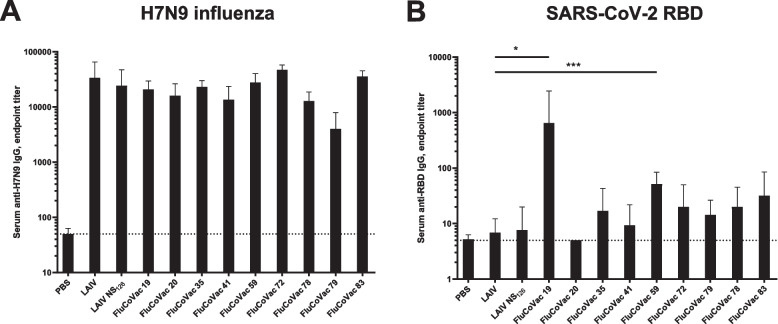
Serum IgG antibody response to H7N9 influenza virus (**A**) and to SARS-CoV-2 RBD (**B**) in BALB/c mice immunized with experimental vaccine strains on day 21 post second immunization (day 42 total). Data from 3 experiments are summarized on the graph. **A** titers of IgG anti-influenza antibodies in sera of immunized animals significantly differ from titers of anti-influenza IgG antibodies from PBS group (statistically significant for all groups, *p*<0.05, Kruskal-Wallis test, post-hoc Dunn’s test, not shown on the graph). **B** (*) *p*<0.05 ANOVA with post-hoc Dunnet’s test, (***) *p*<0.005, Kruskal-Wallis test with post-hoc Dunn’s test

Nevertheless, despite weak virus replication in the mouse respiratory tract, all recombinant vaccine viruses induced high levels of influenza virus-specific serum IgG antibodies three weeks after the second immunization (Fig. 6A). These data indicate that influenza viruses successfully infected target cells and the viral antigens were presented to the mouse immune system. However, rather weak responses were detected to the RBD antigen (Fig. 6B): on day 42 of the study, significant response to RBD was detected only in mice immunized with FluCoVac19 (*p*=0.027, ANOVA with post-hoc Dunnet’s test) and FluCoVac59 (*p*=0.0009, Kruskal-Wallis test with post-hoc Dunn’s test). Levels of serum antibodies in the other groups were not significantly different from control LAIV group. Low responses were registered in several animal sera in the LAIV and LAIV NS_126_ groups, which could be due to the binding of cross-reactive antibodies with low affinity to the RBD protein.

Importantly, the sera of immunized mice were unable to neutralize live SARS-CoV-2 infection in vitro (data not shown), suggesting that the levels of induced anti-RBD antibodies were insufficient to inhibit virus replication in Vero cells under our conditions. Of note, the MN method used in our study has lower sensitivity than the PRNT assay which is used in most studies.

### Assessment of the selected FluCoVac-19 vaccine candidate in Syrian hamsters

**Fig. 7:**
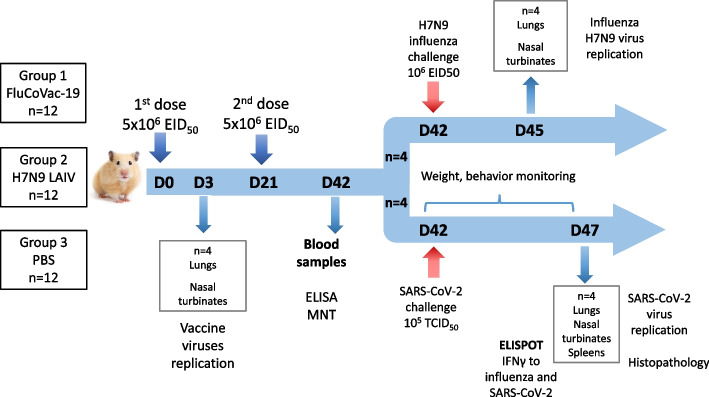
The scheme of the experiment on assessment of safety, immunogenicity and protective potential of the FluCoVac-19 in Syrian hamsters. D – days of the experiment

Because the FluCoVac-19 vaccine candidate demonstrated high immunogenic potential against both influenza and SARS-CoV-2 antigens, this variant was selected for further evaluation in Syrian hamsters. For the assessment of infectivity, immunogenicity and protective activity against influenza and SARS-CoV-2 infections, groups of 12 animals were immunized twice with the recombinant virus and the LAIV vector control at a dose of 5×10^6^ EID_50_, twice with a tree-week interval. A group of control animals received PBS (Fig. 7).

**Fig. 8:**
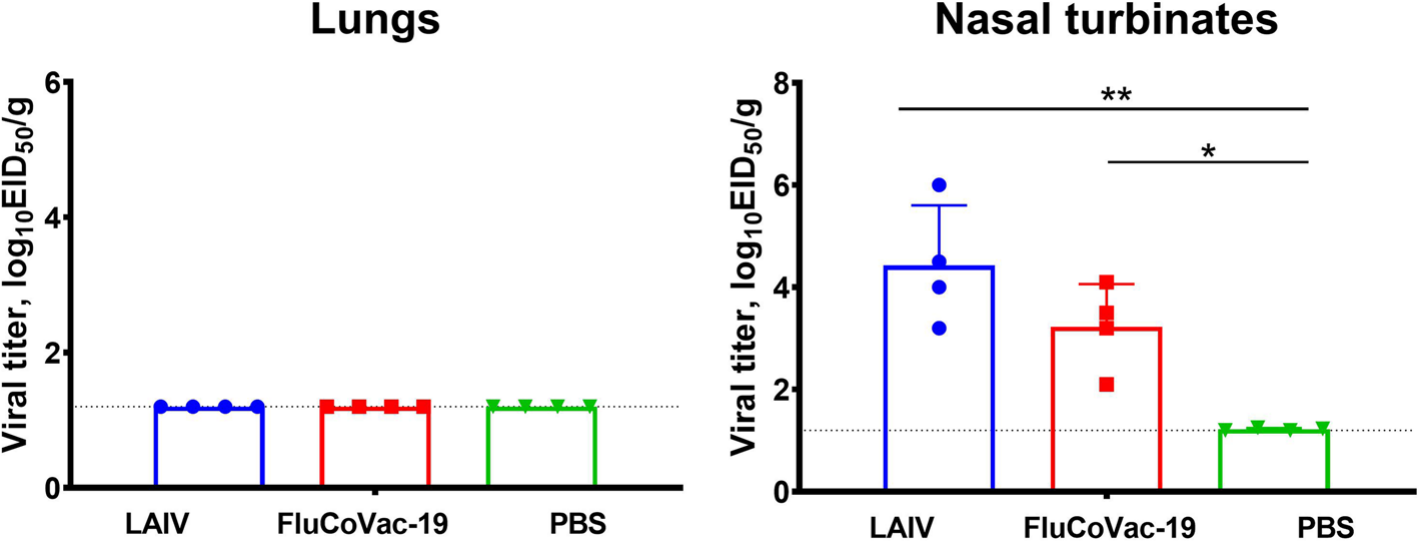
Replication of FluCoVac-19 and control H7N9 LAIV virus in the respiratory tract of Syrian hamsters on Day 3 after immunization. Animals were i.n. immunized with 5×10^6^ EID_50_ of each virus and viral titers in the lungs and in the nasal turbinates (*n*=4) were determined on day 3 post inoculation. Data were analyzed by one-way ANOVA with Tukey’s post-hoc multiple analyses test. *—*p* < 0.05; **—*p* < 0.01; ***—*p* < 0.001

Three days after the first immunization, four animals from each group were humanly euthanized to assess influenza virus replication in the respiratory tract. Infectious titers were determined by titration of tissue homogenates in eggs, with the limit of virus detection 1.2 lgEID_50_. As expected, no virus replication was observed in the lungs of immunized hamsters, confirming the attenuated phenotype of the LAIV virus and the FluCoVac-19 (Fig. 8). In contrast, both viruses replicated efficiently in nasal turbinates, reaching mean titers of 4.4 and 3.2 lgEID_50_/g for the LAIV and FluCoVac-19 viruses, respectively (Fig. 8).

**Fig. 9:**
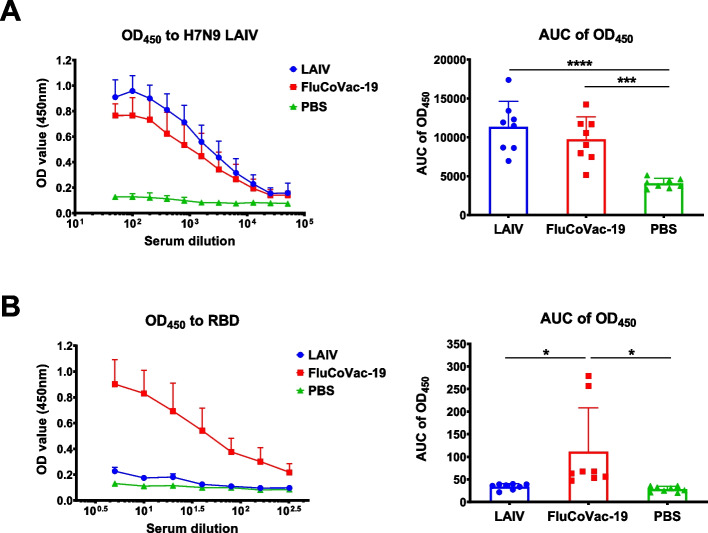
Serum antibody immune responses in Syrian hamsters immunized with FluCoVac-19 experimental vaccine. Syrian hamsters were twice immunized with 5×10^6^ EID_50_ of H7N9 LAIV or FluCoVac-19 at 3-week intervals; sterile PBS was used as a control. Sera were collected 3 weeks after the 2^nd^ dose and assessed by ELISA against whole influenza virus antigen (**A**) or against recombinant RBD protein (**B**). Data were analyzed by one-way ANOVA with Tukey’s post-hoc multiple analyses test. *—*p* < 0.05; ***—*p* < 0.001; ****—*p* < 0.0001

Serum antibody immune responses were measured on day 21 after the second dose by ELISA against whole H7N9 whole influenza virus or recombinant RBD protein. Although the levels of anti-influenza IgG antibodies were slightly lower in the FluCoVac-19 group than in the LAIV control group, these differences were not statistically significant (Fig. 9A). These data indicate that the insertion of the RBD 194 fragment into the HA molecule of the LAIV strain did not impact the overall immunogenicity of the vaccine relative to the influenza virus antigens. Importantly, a significant increase in the anti-RBD IgG antibody levels was found only in animals immunized with the recombinant vaccine candidate (Fig. 9B). Notably, there was a variation in the immunogenicity of the FluCoVac-19 vaccine, as some animals had robust RBD-specific responses, whereas others responded rather weakly to this target antigen (Fig. 9B).

**Fig. 10:**
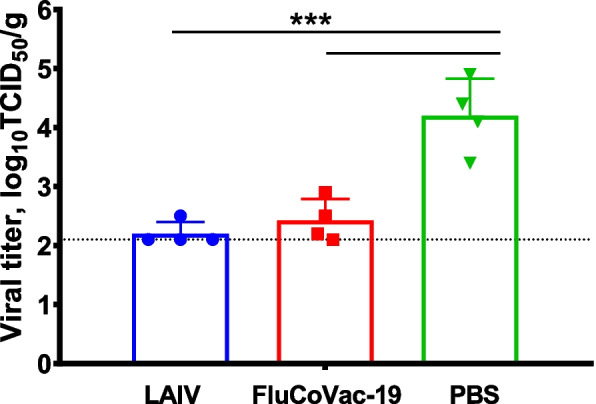
Replication of Sh/PR8 influenza virus in the respiratory tract of Syrian hamsters on day 3 after challenge with influenza virus. Animals were twice immunized with each virus and the challenge influenza virus Sh/PR8 was intranasally inoculated on day 21 after the second dose. Three days post challenge, viral pulmonary titers were determined by titration of tissue homogenates on MDCK cells. Data were analyzed by one-way ANOVA with Tukey’s post-hoc multiple analyses test. *—*p* < 0.05; **—*p* < 0.01; ***—*p* < 0.001

Immunized hamsters (*n*=4) were challenged with Sh/PR8 influenza virus 3 weeks after the second immunization. On day 3 post challenge, a significant reduction in viral pulmonary titers were observed in both LAIV and FluCoVac-19 groups, compared to the hamsters administered with PBS (Fig. 10). These data indicate that the anti-influenza protective immunity was not affected by the modification of the LAIV genome by incorporating a foreign antigen into its HA protein.

The remaining four immunized animals in each group were subjected to SARS-CoV-2 challenge. On Day 42 of the study, hamsters were i.n. infected with 10^5^ TCID_50_ of Wuhan (D614G) SARS-CoV-2 virus. The body weight and clinical symptoms of the disease were monitored for 5 days after challenge. At this time point, the animals were euthanized and respiratory tissues were collected for viral load determination and for the histopathological evaluation. In addition, spleens were harvested, and the numbers of IFNγ-secreting cells in isolated splenocytes were assessed by ELISPOT assay.

**Fig. 11:**
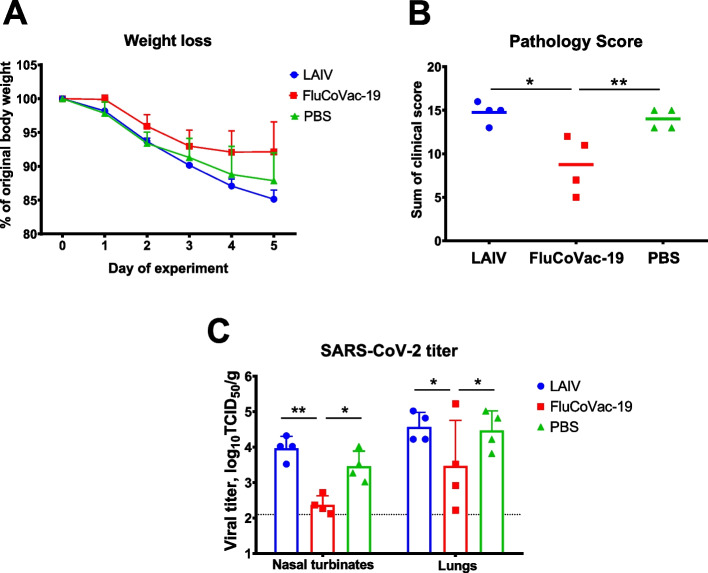
Protective activity of the FluCoVac-19 experimental vaccine in Syrian hamster model of SARS-CoV-2 infection. Syrian hamsters were immunized twice with 5×10^6^ EID_50_ of H7N9 LAIV or FluCoVac-19 at 3-week intervals; sterile PBS was used as a control. Three weeks after the 2^nd^ dose animals were challenged with 10^5^ TCID_50_ of Wuhan (D614G) SARS-CoV-2 virus. **A** Body weight monitoring during five days post challenge. **B** Sum of pathology scores over the challenge phase. **C** SARS-CoV-2 virus titer in lung tissue at day 5 after challenge, assessed by titration in Vero cells. Data were analyzed by one-way or two-way ANOVA with Tukey’s post-hoc multiple analyses test. *—*p* < 0.05; **—*p* < 0.01

The FluCoVac-19 vaccine provided a detectable level of protection against live SARS-CoV-2, as was manifested by reduced weight loss (Fig. 11A), diminished clinical symptoms (Fig. 11B), and reduced viral titers in the URT and LRT of the animals on day 5 post challenge (Fig. 11C), compared to the PBS and H7N9 LAIV groups. Notably, the protection was not even, since one of four animals in the FluCoVac-19 group shed the virus at the same level as control animals.

**Fig. 12:**
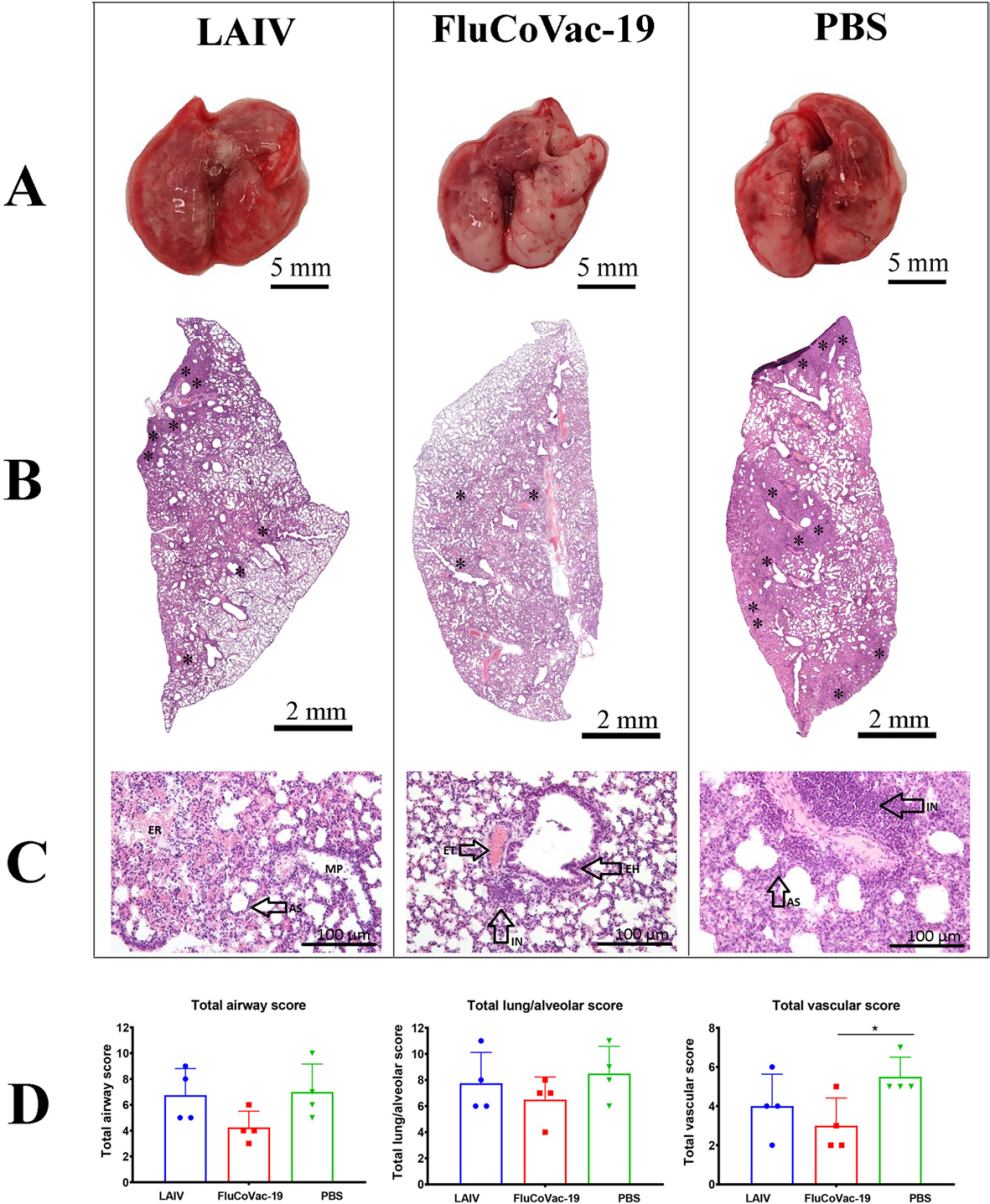
Pathomorphological evaluation of lung tissues of hamsters immunized with FluCoVac-19, or control LAIV, as well as non-immunized animals (PBS group) on day 5 after challenge with SARS-CoV-2. **A** Representative macrographs of the lungs of hamster from each study group. **B** Representative micrographs of the hematoxylin-eosin stained lung sections (magnification ×50): asterisk – foci and/or diffuse mix cell infiltrate **C** Representative micrographs of the hematoxylin-eosin stained lung sections (magnification ×20): AS – alveolar sept, EH – epithelial hyperplasia, ET – endothelial cells, ER – erythrocytes, IN – infiltration cells, MP – macrophages. **D** Semi-quantitative analyses of the airway, lung/alveolar and vascular damage. Data were analyzed by one-way ANOVA with Tukey’s post-hoc multiple analyses test. *—*p* < 0.05

Histopathological evaluation of lung tissues revealed only partial protection of FluCoVac-19-immunized animals against SARS-CoV-2-induced induced alveolar and peribronchiolar inflammation, hemorrhages and endothelial dysfunction (Fig. 12 A-C). In general, the LAIV vector and the chimeric vaccine groups did not differ in the sum of the pathomorphological scale scores, with the exception of a slightly lower severity of vascular changes in the FluCoVac-19 group than in the PBS group (Fig. 12D). It should also be noted that there are no gross histopathological changes in the form of cell rupture or detachment. Overall, based on the clinical, virological and pathomorphological evaluations, the FluCoVac-19 vaccine prototype demonstrated moderate degree of protection against challenge with a high dose of virulent SARS-CoV-2. Further studies with other challenge regimens are needed to fully elucidate the protective potential of this bivalent vaccine candidate.

**Fig. 13:**
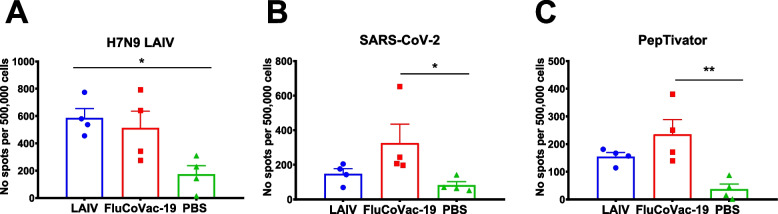
Cell-mediated immune response to influenza and SARS-CoV-2 antigens on day 5 after challenge with SARS-CoV-2 in the splenocytes of immunized Syrian hamsters. Isolated splenocytes were stimulated overnight with **A** H7N9 LAIV purified virus, **B** live SARS-CoV-2 purified virus or with **C** peptide mixture (PepTivator N + S). IFNγ-secreting cells were detected with a Hamster IFN-γ ELISpot Plus kit. Data were analyzed by one-way ANOVA with Tukey’s post-hoc multiple analyses test. *—*p* < 0.05; **—*p* <0.01

We finally assessed cell-mediated immune response to influenza and SARS-CoV-2 antigens by stimulating splenocytes isolated five days post SARS-CoV-2 challenge with influenza and SARS-CoV-2 live viruses, as well as with SARS-CoV-2 peptides (PepTivator (Miltenyi Biotec Bergisch Gladbach, Germany)). Both LAIV and FluCoVac-19 viruses induced influenza-specific IFNγ-secreting cells, although significant difference was noted for the LAIV group only (Fig. 13A). Importantly, significantly higher levels of cytokine-secreting cells were found in the FluCoVac-19 group after stimulation of splenocytes with live SARS-CoV-2 (Fig. 13B) or with PepTivator (Fig. 13C). These data indicate that this vaccine candidate has primed the T-cell arm of immune system of hamsters, along with the RBD-specific antibody immunity. This is in line with the known ability of LAIV viruses to stimulate memory T-cell immune responses after intranasal immunization. Since the inserted RBD fragment is processed along with other influenza virus proteins, T cells that are specific to the epitopes located within RBD, can also be efficiently activated after immunization and provide additional mode of protection against the disease.

## Discussion

This paper describes the development of a bivalent viral-vectored vaccine against SARS-CoV-2 and influenza based on attenuated strain of live influenza vaccine as a vector, with an inserted RBD-based immunogenic fragment of SARS-CoV-2. We investigated a list of modifications of influenza virus by inserting immunogenic material into HA and NS genes using different strategies. Furthermore, we assessed several modifications of the RBD insert that differed by the length and the targeting signals. All rescued candidate vaccine strains were studied in vitro, and immunogenicity was evaluated in a mouse model to select the most promising candidate for further challenge experiments in Syrian hamsters.

Various published works have been thoroughly evaluated to support our choice of the RBD-based antigen design for further incorporation into influenza virus genome. The most important issue to consider when developing vaccines that use RBD is antigen design – e.g. which fragment will be inserted into the vaccine, as this will affect the spatial structure of the fragment, as well as the repertoire of the T-cell epitopes in the final antigen structure. Boundaries of the RBD mapped on the reference strain sequence YP_009724390 are indicated as amino acid residues 330-583 of the Spike protein. In five of our constructs exploring various targeting strategies, we tested the prolonged variant of RBD 278 cassette (319-596). This construct was designed by BIOCAD JSC (Russia) [19] and was highly immunogenic as an AAV-vectored vaccine candidate. A similar CHO-expressed RBD_319-591_ fragment induced virus-neutralizing antibody response [56].

In a study by Lan et al. [32], RBD is indicated at residues 319-541, which corresponds to the RBD 223 insert used in our study in variants FluCoVac-20, 35 and 41. Recombinant protein vaccines [57] and influenza VLP-based vaccines [5] based on the RBD_319-541_ fragment were shown to be immunogenic for animals and correctly folded, which was established in tests with COVID-19 convalescent sera. Similar RBD_319-545_ fragment was expressed in a baculovirus system [58].

The resolved structure of RBD complex with the ACE2 receptor indicated that residues 333-527 are involved in interactions with the receptor and the structure formation [32]. Thus, this is minimal variant of RBD that was tested in our study as the RBD 194 fragment, which was embedded in the influenza HA molecule, thus producing the FluCoVac-19 candidate. Specifically, we used residues 333 to 526, because the P527 connected to the linker could result in artificial folding of the RBD fragment fused to the HA1 subunit via the (G_4_S)_2_ linker. A similar truncated structure of RBD domain (331-524) was studied as an immunogenic mRNA vaccine [59]. Prolonged variants were also successful: recombinant RBD_330-532_ fused to the Fc-fragment of IgG1 induced the formation of RBD-specific neutralizing antibodies in mice [60]; in addition, the RBD_331-531_ was shown to be immunogenic in an influenza-vectored vaccine [11, 12].

In this study, we used two fundamentally different strategies to design the LAIV-RBD recombinant viruses: incorporation of RBD-encoding material into the HA ORF, and insertion of RBD-encoding cassettes into truncated NS1 gene. The first strategy should lead to the exposure of a large number of the RBD copies on the surface of the virion as a part of the chimeric HA glycoprotein; however, the size of the insert is limited in such designs, since too large insertion can significantly reduce virus infectivity [31]. Of the two RBD variants incorporated into the HA molecule (RBD 194 and RBD 223), only the RBD 194 was proven successful since this variant indeed expressed the inserted fragment as a stable fusion HA+RBD protein. Strikingly, the RBD 223-based variant failed to express the target antigen as a fusion HA+RBD protein, although high level of RBD protein expression was detected in virus-infected cells. Since the correct folding of the RBD_319-541_ alone was confirmed in other studies [5, 57], we assumed that the RBD fragment could be cleaved post-translationally by some proteases. The RBD 194-based variant, FluCoVac-19, was proved to be safe and immunogenic when administered intranasally to Syrian hamsters, and the induced immune responses to the influenza and SARS-CoV-2 antigens afforded combined protection of animals against both infections.

Similar strategies exploiting incorporation of RBD into influenza virus particle outer membrane was used by other scientific groups. A non-replicating virus vector lacking the HA ORF, with modified HA and M genetic segments was studied by Koonpaew et al. [14]. The cassette encoding RBD_325-532_ with tPA signal sequence and HA transmembrane and cytoplasmic domains was inserted under influenza segment 4 UTRs. The vaccine induced SARS-CoV-2 neutralizing antibodies and anti-influenza serum IgG response, but the T-cell responses to influenza HA were not remarkable due to the non-replicating vector. The other limitation of this vaccine is the requirement of special HA-expressing cells for virus production. In another development, a replicating influenza virus encoded the RBD fragment which was inserted in-frame with HA protein via the P2A autocleavage site [15]. The incorporation of RBD into the membrane is afforded by the addition of cytoplasmic tail and transmembrane domain of NA. Notably, the immunization with inactivated virus did not protect animals against SARS-CoV-2 challenge, whereas using this virus as a live vaccine significantly increased its protective potential. This is consistent with the significant impact of the local immune response and T-cell-mediated immunity on SARS-CoV-2 protection [61, 62]. The peculiarity of live virus-vectored vaccines is the direct stimulation of the antiviral T-cell response, and intranasal application provides effective stimulation of local immunity. It should be noted that none of the influenza virus vectors used by other research groups have been used in a licensed influenza vaccine product marketed for human use, which is in contrast to the Len/17-based LAIV platform used in our study.

Using the second strategy with modification of NS, we designed several variants of NS gene modifications and cassette targeting. We were unable to detect RBD expression in MDCK cells infected with any NS-based prototype, as well as the induction of RBD-specific antibodies in immunized mice. A similar design was used in the dNS1-RBD vaccine developed in China [9]. In this vaccine, the cold-adapted A/California/04/2009 (H1N1pdm09) virus lacking NS1 was used as a vector [63], and the fragment encoding RBD_316-550_ with B2M signal peptide and foldon with the V5 tag was incorporated into the NS gene instead of the NS1 ORF [9]. The results of Phase I and II of clinical trials demonstrated that this vaccine was safe and well-tolerated [10]. Importantly, this vaccine did not induce neutralizing antibody response in mice, and the levels of anti-RBD serum IgG antibodies were comparable to our results obtained for the FluCoVac-19 candidate, whereas protective efficacy of the vaccine was demonstrated in Syrian hamsters using the virus transmission model. In our experiments, we use direct virus inoculation model that allows the higher dose of the virus to enter the airways simultaneously. The growth characteristics of the recombinant dNS1-RBD virus were decreased compared to those of the non-modified influenza virus. In our experiments, viruses with modified NSs also had decreased titers compared to the classical LAIV, whereas no negative effect of the RBD insert on viral growth characteristics was observed compared to the vector virus with truncated NS. The detailed analysis of the immune response on dNS1-RBD demonstrated high importance of the cell-mediated immunity, especially in the lungs. Therefore, in our further experiments, we plan to evaluate T-cell immunity to the NS-based chimeric viruses, with special attention to the lung-localized memory T cells [64].

One of the reasons for the lower immunogenicity of the RBD-based recombinant influenza viruses based on A/Leningrad/17 backbone compared to the other backbones could be the inability of the Len/17-based viruses to efficiently replicate in the mouse respiratory tract, especially with NS1 modifications [17]. For example, A/PR/8/34-based influenza viruses, even in the case of truncated NS1, replicate well in the lungs [65], thus producing higher levels of virus-specific serum antibodies. We suppose that the NS-based LAIV-RBD vaccine prototypes can be further improved, because this strategy seems to be effective in designing vectored vaccines against other diseases [9, 12, 22, 35, 64, 66], and also because this strategy is promising in terms of annual updates of seasonal influenza vaccines.

Our study has several limitations. In this study, we did not assess the durability of the antibody responses and the maintenance of protective effect of immunization with FluCoVac-19 vaccine candidate. However, it is known that the effect of LAIV immunization is mediated by a complex of immunological barriers, including long-lived tissue resident memory cells, and it lasts for at least one year [67]. The persistence of immune responses were assessed for the dNS1-based SARS-CoV-2 chimeric vaccine, and they lasted for at least 3 months [9]. We studied the protective effect of only one vaccine candidate with established expression of RBD protein and pronounced immunogenicity in pilot animal experiments, since the presence of correctly folded protein is a prerequisite of the induction of functional antibody responses to the target spatial epitopes. Unexpectedly, we couldn’t confirm the expression of RBD in NS-based candidates, and the exact reason for this was not yet established; it could be artificial folding of the expressed protein or quick proteasome degradation of the RBD-based construct. In this case, T-cell immunity could have provided protection even in the absence of detectable RBD expression. We plan to study this in details in our future experiments.

The strategy we used provided protection of the animals challenged with homologous strains of influenza and SARS-CoV-2 viruses. Seasonal influenza vaccines for human use are currently updated twice a year, before the epidemic seasons in Nothern and Southern hemispheres. For SARS-CoV-2, the updates are also of current interest, because of high mutations rate in circulating omicron subvariants. The development of the LAIV-based SARS-CoV-2 vaccine which can be regularly updated to make it a promising bivalent vaccine for influenza and SARS-CoV-2 prevention is a major focus of our future research.

## Conclusions

In this study, we developed a panel of RBD-based LAIV virus vector-based bivalent vaccine candidates for combined protection against influenza and COVID-19. Based on the results of the in vitro assessment and pilot experiment in BALB/c mice, we selected FluCoVac-19 vaccine candidate for challenge experiments. This candidate encodes modified HA protein that includes the RBD fragment (residues 333-526) on the N-terminus of the HA1 subunit connected via the (G_4_S)_2_ linker. The RBD expression was confirmed by western-blot analysis of purified influenza virus and by sandwich ELISA of virus-infected MDCK cell lysates. Intranasal immunization induced serum IgG antibody responses to the whole influenza virus and to the RBD protein both in mice and Syrian hamsters. This vaccine candidate protected Syrian hamsters against challenge with H7N9 influenza virus and SARS-CoV-2, thus confirming that this strategy is promising for the vaccine development for the combined prevention of influenza and COVID-19.

### Supplementary Information

**Supplementary Material 1.**

**Supplementary Material 2.**

## Data Availability

No datasets were generated or analysed during the current study.
