# Supplementary figures and images for: Expression of the SARS-CoV-2 receptor-binding domain by live attenuated influenza vaccine virus as a strategy for designing a bivalent vaccine against COVID-19 and influenza
Source: Virol J. 2024 Apr 9;21:82. doi: 10.1186/s12985-024-02350-w (PMC11003101; doi:10.1186/s12985-024-02350-w)

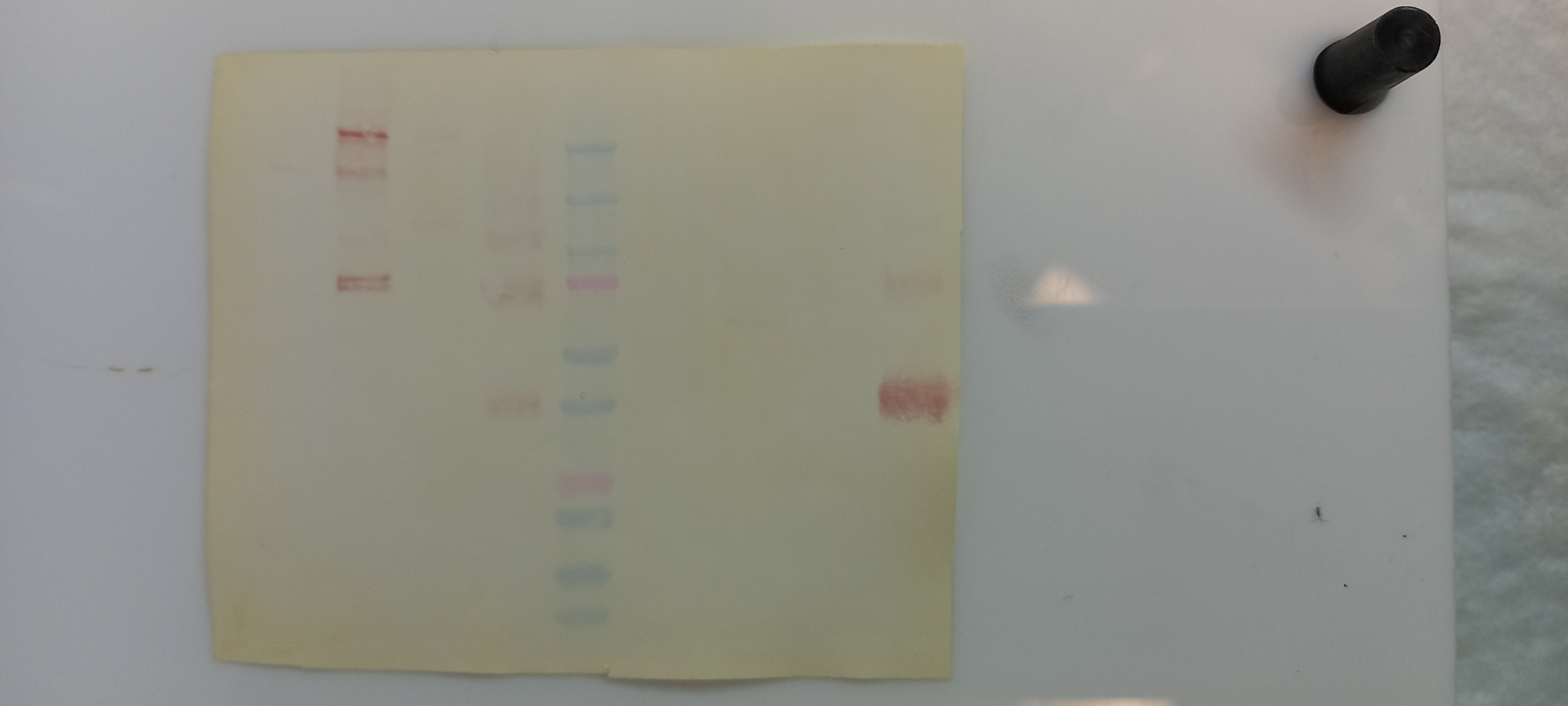

Supplement: Supplementary file 1 — Supplementary Material 1. [file 12985_2024_2350_MOESM1_ESM.jpg]

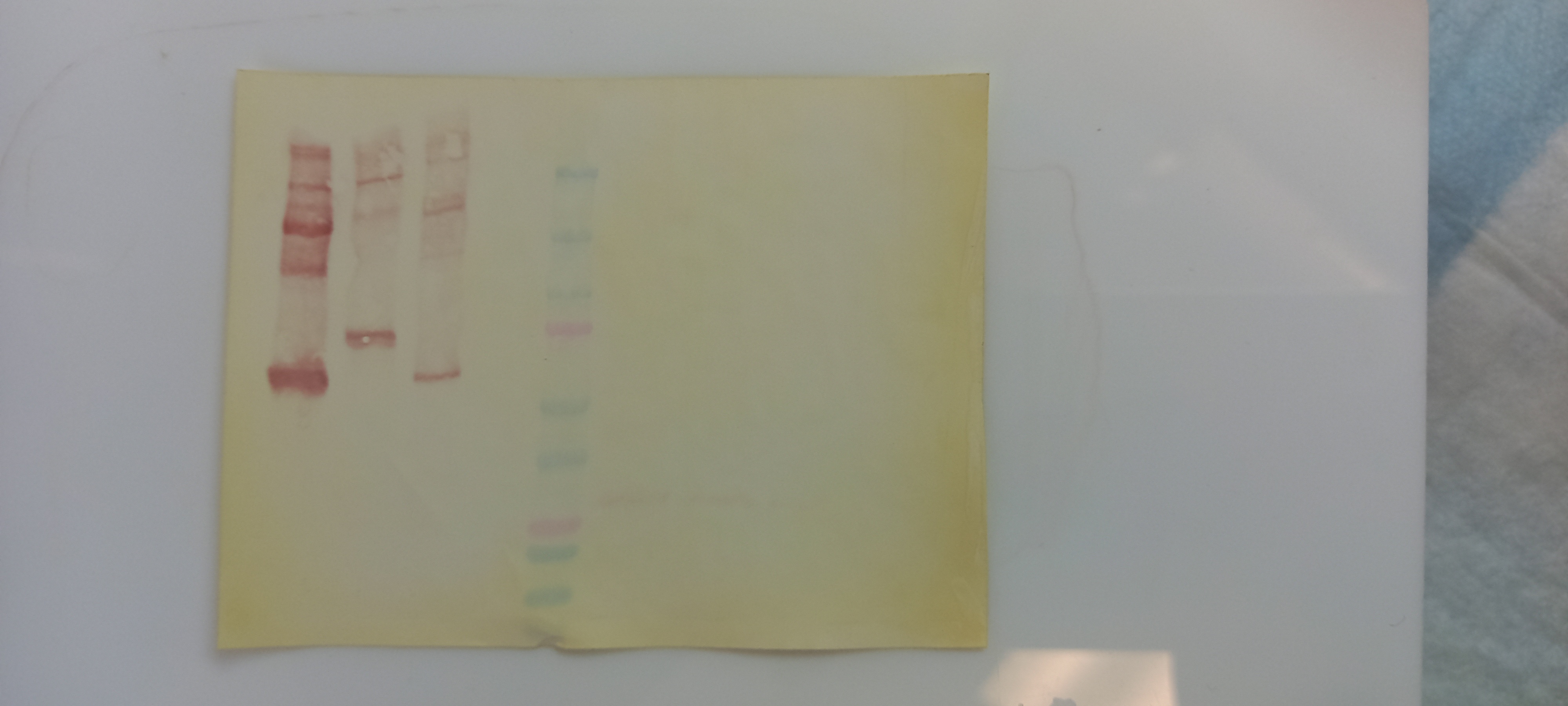

Supplement: Supplementary file 2 — Supplementary Material 2. [file 12985_2024_2350_MOESM2_ESM.jpg]
